# Charged Polypeptide Tail Boosts the Salt Resistance of Enzyme-Containing Complex Coacervate Micelles

**DOI:** 10.1021/acs.biomac.1c01466

**Published:** 2022-01-19

**Authors:** Riahna Kembaren, Adrie H. Westphal, Marleen Kamperman, J. Mieke Kleijn, Jan Willem Borst

**Affiliations:** † Physical Chemistry and Soft Matter, Wageningen University & Research, Stippeneng 4, 6708 WE Wageningen, The Netherlands; ‡ Laboratory of Biochemistry, Microspectroscopy Research Facility, Wageningen University & Research, Stippeneng 4, 6708 WE Wageningen, The Netherlands; § Polymer Science, Zernike Institute for Advanced Materials, University of Groningen, Nijenborgh 4, 9747 AG Groningen, The Netherlands

## Abstract

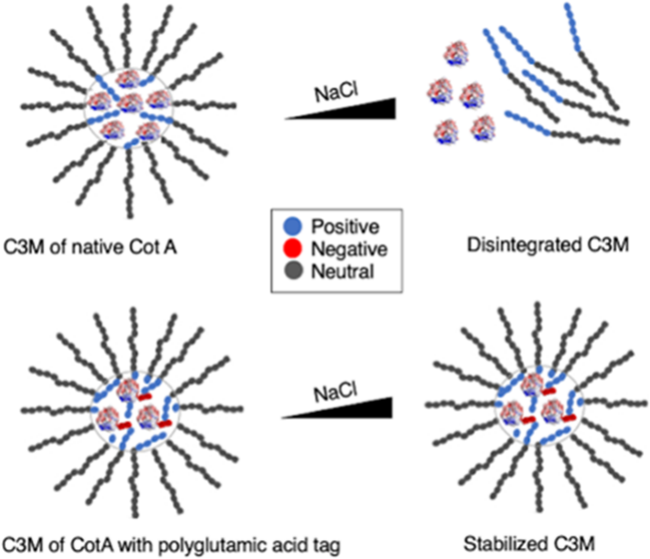

Encapsulation of proteins can have advantages for their protection, stability, and delivery purposes. One of the options to encapsulate proteins is to incorporate them in complex coacervate core micelles (C3Ms). This can easily be achieved by mixing aqueous solutions of the protein and an oppositely charged neutral-hydrophilic diblock copolymer. However, protein-containing C3Ms often suffer from salt-inducible disintegration due to the low charge density of proteins. The aim of this study is to improve the salt stability of protein-containing C3Ms by increasing the net charge of the protein by tagging it with a charged polypeptide. As a model protein, we used CotA laccase and generated variants with 10, 20, 30, and 40 glutamic acids attached at the C-terminus of CotA using genetic engineering. Micelles were obtained by mixing the five CotA variants with poly(*N*-methyl-2-vinyl-pyridinium)-*block*-poly(ethylene oxide) (PM2VP_128_-*b*-PEO_477_) at pH 10.8. Hydrodynamic radii of the micelles of approximately 31, 27, and 23 nm for native CotA, CotA-E20, and CotA-E40, respectively, were determined using dynamic light scattering (DLS) and fluorescence correlation spectroscopy (FCS). The encapsulation efficiency was not affected using enzymes with a polyglutamic acid tail but resulted in more micelles with a smaller number of enzyme molecules per micelle. Furthermore, it was shown that the addition of a polyglutamic acid tail to CotA indeed resulted in improved salt stability of enzyme-containing C3Ms. Interestingly, the polyglutamic acid CotA variants showed an enhanced enzyme activity. This study demonstrates that increasing the net charge of enzymes through genetic engineering is a promising strategy to improve the practical applicability of C3Ms as enzyme delivery systems.

## Introduction

Protein encapsulation can shield valuable proteins in denaturing or degrading environments and also offers the possibility of targeted release, which is beneficial for many applications, especially for protein drug delivery. Many methods have been developed to encapsulate biomacromolecules, and one of the most promising is the use of complex coacervate core micelles (C3Ms).^1−3^ Encapsulation of proteins into C3Ms can be achieved by mixing a charged protein with a diblock copolymer, which has a countercharged part and a neutral-hydrophilic part.^1,3−6^ When these two components are mixed in a low ionic strength buffer solution, the oppositely charged parts bind electrostatically to form a complex coacervate core. The neutral-hydrophilic part of the diblock copolymer acts as shell/corona that ensures that this core–shell structure remains soluble in an aqueous solution. The formation of C3Ms is also driven by the entropy gain upon the release of counterions, which makes this packing system sensitive to high ionic strength. Therefore, a high salt environment will result in the disintegration of protein-containing C3Ms. Since proteins have a pH-dependent charge, this packing system is also sensitive to pH.^1,6−10^

One way to improve the stability of protein-containing C3Ms is by increasing the net charge of the protein using post-translational chemical modification or genetic engineering.^9−15^ Bioconjugation approaches allow the addition of negative charges to the protein, for example, by coupling the protein with poly(acrylic acid) (PAA).^16,17^ Acetylation of lysine residues using acetic anhydride has also been shown to result in protein variants with an increased negative charge.^18^ However, this acetylation approach lacks bioconjugation specificity and results in heterogeneous labeling.^9^ Alternatively, the net charge of proteins can be changed by the addition of a genetically engineered charged polypeptide tag at a specific site of the protein.^9,13,42,43^ Genetic engineering thus allows us to produce supercharged recombinant proteins with a high yield and with precise composition.^9^ Kapelner and Obermeyer demonstrated that the addition of an anionic polypeptide tag at the C-terminus of green fluorescent protein (GFP) promoted coacervation at higher salt concentrations.^13^

**Figure 1:**
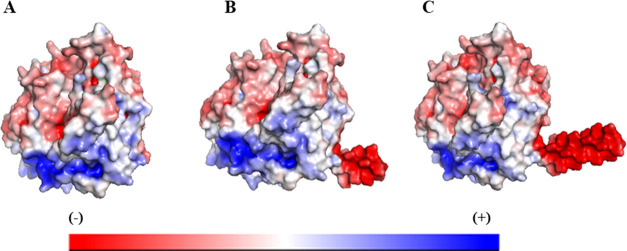
Comparison of electrostatic potentials at the molecular surface of three CotA variants. (A) Native CotA (CotA wild type), (B) CotA with additional 10 glutamic acid tags (CotA-E10), and (C) CotA with additional 20 glutamic acid tags (CotA-E20). Color surface overlay indicates the electrostatic potential in a scale from negative (red), neutral (white), and positive potential (blue). This figure was created using the default parameters of the PyMOL APBS Tools plugin in PyMol_2.3.4 software.

The aim of this study is to improve the stability of protein-containing C3Ms by increasing the net charge of the protein by the addition of a charged polypeptide tag. Genetic engineering enables us to add tags or specific sequences after the start codon (N-terminus part of a protein) or before the stop codon (C-terminus part of a protein) or in the exposed surface loops.^19,43^ In comparison to N-terminus or loop tagging, introducing a tag at the C-terminus has generally less effect on protein folding and its biological function.^20,21^ We used CotA laccase as a model protein and added different polyglutamic acid tags at the C-terminus of the enzyme (see Figure 1). C3Ms were formed in a buffer solution of pH 10.8. As described in our previous paper,^22^ this pH was chosen to establish a sufficient negative charge on native CotA to form C3Ms and to eliminate the effect of the positively charged patch on its surface.

The salt stability of differently charged CotA-containing C3Ms was determined using dynamic light scattering (DLS) and fluorescence correlation spectroscopy (FCS). The enzyme activity of the various CotA variants was tested before and after encapsulation.

## Experimental Section

### Materials

The diblock copolymer poly(2-vinyl pyridine)_128_-*block*-poly(ethylene oxide)_477_ (P2VP_128_-*b*-PEO_477_) (*M*_w_/*M*_n_ = 1.1, *M*_n_ = 34.5 kg/mol) was obtained from Polymer Source Inc. After quaternization with iodomethane,^23^ the quaternization degree of this diblock copolymer was 73% (measured by ^1^H NMR) (Figure S1, Supporting Information).^24^ All primers for gene modification were obtained from Integrated DNA Technologies (IDT). DNA purification kits (miniprep, PCR purification, and gel purification) were purchased from Thermo Fisher Scientific. Lysogeny broth and agar medium for the growth of *Escherichia coli* were purchased from Duchefa Biochemie. Columns for enzyme purification (SP-Sepharose FF, Q-Sepharose FF, Superdex 200, and Heparin Sepharose) were obtained from GE Healthcare. Alexa Fluor 488 C5 maleimide obtained from Thermo Fisher Scientific was used to fluorescently label the CotA variants. To remove the unreacted label, a Biogel-P6DG gel filtration column obtained from Bio-Rad was used. A Pierce bicinchoninic acid (BCA) protein assay kit for the determination of total protein concentration was obtained from Thermo Fisher Scientific. The substrate 2,2′-azino-bis(3-ethylbenzothiazoline-6-sulfonic acid) (ABTS) for the activity assay was purchased from Sigma-Aldrich.

### Modification of the CotA Gene and Cloning Using the Seamless Ligation Cloning Extract (SLiCE) Method

To clone the gene of our model protein, we used the SLiCE method. This method is based on *in vitro* recombination of short regions of homologies (about 15–52 base pair overlap sequences between target DNA fragment and vector) in bacterial cell extracts. It is an easy and inexpensive cloning method compared to conventional cloning methods. The CotA gene was modified with primers, which contained an overlap part with the pBAD vector sequences and glutamic acid sequences of different lengths, i.e., 10 (CotA-E10), 20 (CotA-E20), 30 (CotA-E30), and 40 (CotA-E40). Native CotA was also mutated at position 313 where a serine was replaced by a cysteine (CotA-S313C). This mutation allows us to specifically label the enzyme with Alexa Fluor 488 C5 maleimide. After the S313C mutation, this CotA gene was used as a template for additional glutamic acid tags with a length of 20 (CotA-S313C-E20) and 40 (CotA-S313C-E40). Agarose electrophoresis results show that the genetic engineering of CotA with several additional units of glutamic acids tags was successful (Figures S2–S4, Supporting Information). These modified genes were then cloned into the pBAD vector. The cloned genes were transformed in *E. coli* DH5α, and the bacteria were grown on ampicillin-containing lysogeny broth (LB) media. After growing for 1 day, colonies were checked by colony-PCR and the presence of correct inserts was confirmed by the digestion of purified plasmids using restriction enzymes *Nde*I and *Hin*dIII. The positive plasmids were sent for sequencing to Macrogen Europe.

### CotA Production and Purification

The CotA and modified CotA genes were transformed into *E. coli* Rosetta cells. Single colonies were picked and grown overnight in LB medium containing 100 μg/mL ampicillin. The overnight culture was inoculated on a 500 mL LB medium, and the cells were grown until an optical density of 0.6–0.8. CotA laccase expression was induced by adding 0.15% l-arabinose and 0.25 mM CuSO_4_, and the cells were grown for 20 h at 25 °C. For CotA-S313C, CotA-S313C-E20, and CotA-S313C-E40, the induction was done by only adding 0.15% l-arabinose (without copper salt solution) since copper can promote the oxidation of free sulfhydryl of the cysteine. Native CotA, CotA-S313C, and CotA-E10 were purified using cation-exchange chromatography (cIEX using an SP-Sepharose FF column) and gel filtration chromatography (Superdex 200 column). CotA-E20, CotA-E30, and CotA-S313C-E20 were purified using anion-exchange chromatography (aIEX using a Q-Sepharose FF column) followed by gel filtration chromatography (Superdex 200 column). CotA-E40 and CotA-S313C-E40 were purified using anion-exchange chromatography (aIEX using a Q-Sepharose FF column) followed by gel filtration chromatography (Superdex 200 column) and Heparin Sepharose column chromatography.

Specific fluorescence labeling of the enzyme was performed by mixing the enzyme with Alexa Fluor 488 C5 maleimide with a molar ratio of about 1:10 followed by incubation at 4 °C in the dark overnight. To remove the unreacted label, the mixture was loaded to a Biogel-P6DG gel filtration column using buffer 20 mM Tris–HCl containing 10 mM NaCl. The fractions that showed fluorescence and contained protein were pooled and concentrated using a spin filter concentrator. Next, the labeled CotA was further purified on a gel filtration column (Superdex 200 column). The fractions that showed absorption at both 280 and 490 nm were collected and concentrated using a spin filter concentrator. The purity of CotA was analyzed by SDS-PAGE.

### CotA Activity Measurement

We investigated the effect of polyglutamic acid tags on the CotA C-terminus by measuring the enzyme activity using 2,2′azino-bis-(3-ethylbenzothiazoline-6-sulfonic acid) (ABTS) as a substrate for the assay. The activity of the CotA variants before and after C3M formation has been measured. We used 1.0 mM ABTS in 100 mM sodium acetate buffer at pH 4.4. CotA will oxidize ABTS resulting in a green-colored cationic radical (ABTS^•+^), which can be detected by measuring the absorption at wavelength 420 nm (ε = 36 000 M^–1^ cm^–1^). The relative activity was defined as the ratio between the specific activity of modified CotA and the specific activity of native CotA and expressed as a percentage.^25−27^ The total protein concentration was determined using the BCA protein assay.

### Formation of C3Ms Containing CotA and CotA Variants and Determination of Their Salt Stability

Enzyme solutions and solutions of the polymer PM2VP_128_-*b*-PEO_477_ were prepared separately in a 10 mM sodium carbonate buffer at pH 10.8. All solutions were filtered through 0.2 μm poly(ether-sulfone) membrane syringe filters (Advanced Microdevices Pvt. Ltd.). Mixtures of these solutions were prepared with the same final enzyme concentration but increasing concentrations of the diblock copolymer and stored at room temperature overnight before measurement. The mixed ratio composition *F*^–^ is calculated using the equation , where *n*^–^ refers to the concentration of the net negative charge on the enzyme molecules and *n*^+^ refers to the positive charge concentration on the diblock copolymer. PM2VP_128_-*b*-PEO_477_ has a charge of about +93 (elementary units). We used software PROPKA 3.1 to calculate the net charge of the various CotA variants from their three-dimensional structure (Figure S5, Supporting Information).^4,5^ This resulted for native CotA laccase in buffer pH 10.8 in a net charge of about −41, while the genetically engineered CotA-E10, CotA-E20, CotA-E30, and CotA-E40 have net charges of about −51, −61, −71, and −81, respectively.

For salt stability determination, 4 M NaCl solution was titrated to solutions of enzyme-containing micelles, and the effect on the micelles was followed using dynamic light scattering (DLS) and fluorescence correlation spectroscopy (FCS). For the DLS and FCS measurements, (each *n* = 3) were performed and these measurements consisted of 10 repetitions of 10 s duration for DLS and 8 repetitions of 20 s duration for FCS. Each of these repetitions were composed of 10 measurements of 10 s for DLS and 8 measurements of 20 s for FCS were done. For FCS measurements, Alexa Fluor 488 labeled enzymes of native CotA, CotA-E20, and CotA-E40 were used. FCS data were analyzed with the software FFS data processor, version 2.3 (Scientific Software Technologies Software Centre, Belarus), using a three-dimensional (3D) diffusion including the triplet state model.^4,5,22^

### Dynamic Light Scattering

DLS was performed with an ALV-LSE 41/CGS-8F goniometer instrument equipped with a DPSS laser operating at 660 nm, and the laser power used was 200 mW. Measurements of scattering intensity, hydrodynamic radius (*R*_h_), and polydispersity index (PDI) were performed at a fixed scattering angle of 90°. The shape of the C3Ms was determined using multiangle DLS.

The principle of DLS is to measure the fluctuation of scattered light intensity, which occurs when laser light hits the diffusing particles. This fluctuation of scattered light intensity depends on the particle movement caused by Brownian motion.^28^ Tracing this fluctuating intensity over time (*t*) enables us to plot a second-order autocorrelation function as shown in the equation
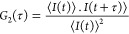
1where *I*(*t*) is the time-dependent scattered light intensity. The field-correlation function of monodisperse particles can be described with an equation featuring the decay rate (Γ) and the passed time (τ)
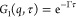
2The relation of the decay rate (Γ) and the diffusion coefficient (*D*) is given by
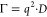
3where *q* is the wave vector, which is defined by the used wavelength (λ_0_), the refractive index of the medium (*n*), and the detection angle (θ) according to the equation
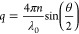
4The hydrodynamic radius (*R*_h_) was calculated from the diffusion coefficient (*D*) obtained from the autocorrelation function by a cumulant fit method and the Stokes–Einstein equation for spherical particles
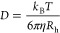
5where *k*_B_ is the Boltzmann constant, *T* is the absolute temperature, and η is the viscosity of the solution. The polydispersity index (PDI) is a representation of the particle size heterogeneity in a sample and is in DLS usually calculated from the width at half-height of the relevant peak in the particle size distribution using the equation
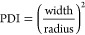
6where the radius is the mean hydrodynamic radii of the peak. Samples with a PDI of 0.0–0.1 are considered to be very monodisperse, while PDI values ranging from 0.1 to 0.4 indicate a moderate polydisperse sample and PDI values >0.4 are indicative for highly polydisperse samples.^29^ While PDI 0.1 is considered to be very monodisperse, values 0.1–0.4 are classified as moderate polydispersity, and >0.4 is considered highly polydisperse.^29^

### Fluorescence Correlation Spectroscopy

FCS was performed using a Leica TCS SP8 X system equipped with a 63× 1.20 NA water immersion objective and a supercontinuum laser. CotA labeled with Alexa Fluor 488 was excited at 488 nm with a pulse frequency of 40 MHz. Fluorescence was detected between 495 and 550 nm using a hybrid detector coupled to a PicoHarp 300 TCSPC module (PicoQuant) with a pinhole setting of 1 Airy unit. FCS data were analyzed with software FFS data processor version 2.3 (Scientific Software Technologies Software Centre, Belarus) using a two-component 3D diffusion model including triplet state.^30^ Rhodamine 110 (diffusion coefficient 4.3 × 10^–10^ m^2^ s^–1^) was used to determine the confocal structure *a* parameter (*a* = ω*_z_*/ω*_xy_*, where ω*_xy_* and ω*_z_* are the equatorial and axial radii of the detection volume, respectively).

In FCS, the fluctuations in fluorescence intensity, resulting from fluorescent particles traversing the confocal detection volume, are recorded over time and used to calculate the autocorrelation function *G*(*t*) as follows
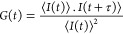
7where *I*(*t*) is the time-dependent fluorescence intensity. After excitation of a fluorophore, transition from the singlet state to the triplet state (intersystem crossing) may occur. Relaxation from the triplet state to the ground state (microsecond time scale) can occur without the emission of photons. This intersystem crossing may lead to an additional component in the autocorrelation function. In the following autocorrelation equation, the triplet state component is included
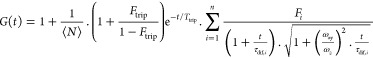
8where ⟨*N*⟩ is the average number of fluorescent particles in the confocal volume, *F*_trip_ is the fraction of molecules in the triplet state, *T*_trip_ is the average time a fluorophore resides in the triplet state, *F*_*i*_ is the fraction of species *i*, τ_dif,*i*_ is the diffusion time of the species *i* in the confocal volume, and ω*_xy_* and ω*_z_* are the equatorial and axial radii of the detection volume, respectively. From the diffusion time, the diffusion coefficient *D* can be calculated using this equation
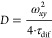
9Subsequently, from *D*, the hydrodynamic radius of the fluorescent particle can be calculated using the Stokes–Einstein relation (eq 5).

## Results and Discussion

### CotA Production and Purification

**Figure 2:**
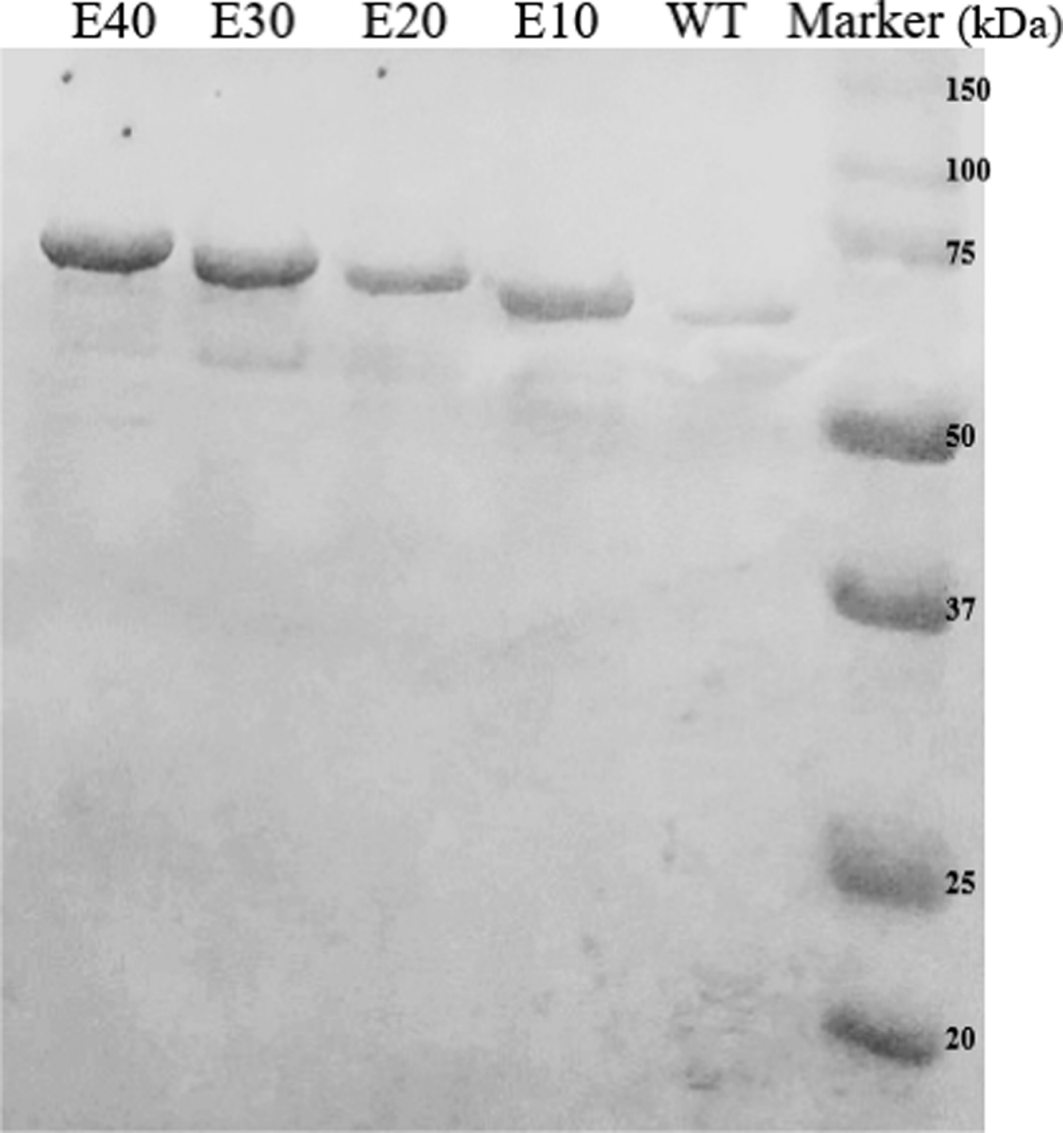
SDS-PAGE of purified CotA-WT, CotA-E10, CotA-E20, CotA-E30, and CotA-E40.

The engineered CotA proteins were overexpressed in the *E. coli* Rosetta strain and purified by a combination of ion-exchange chromatography and size exclusion chromatography. Figure 2 shows the results of an SDS-PAGE of the purified native CotA and the glutamic acid CotA variants. The purified CotA samples are 85–95% pure (analyzed using ImageJ software). The addition of polyglutamic acid tags resulted in a stepwise increase in molecular weight, more precisely from 65 kDa (native CotA) to 66.5 kDa (CotA-E10), 67.9 kDa (CotA-E20), 69.4 kDa (CotA-E30), and 70.9 kDa (CotA-E40). Moreover, the purified native CotA and glutamic acid CotA variants also show an intense blue color as a sign of incorporation of the copper ion (T1 Cu ion)^22,44^ in the enzyme (Figure S6, Supporting Information). From these results, we conclude that the CotA variants are well expressed and purified.

### Encapsulation of CotA and CotA Variants in C3Ms

**Figure 3:**
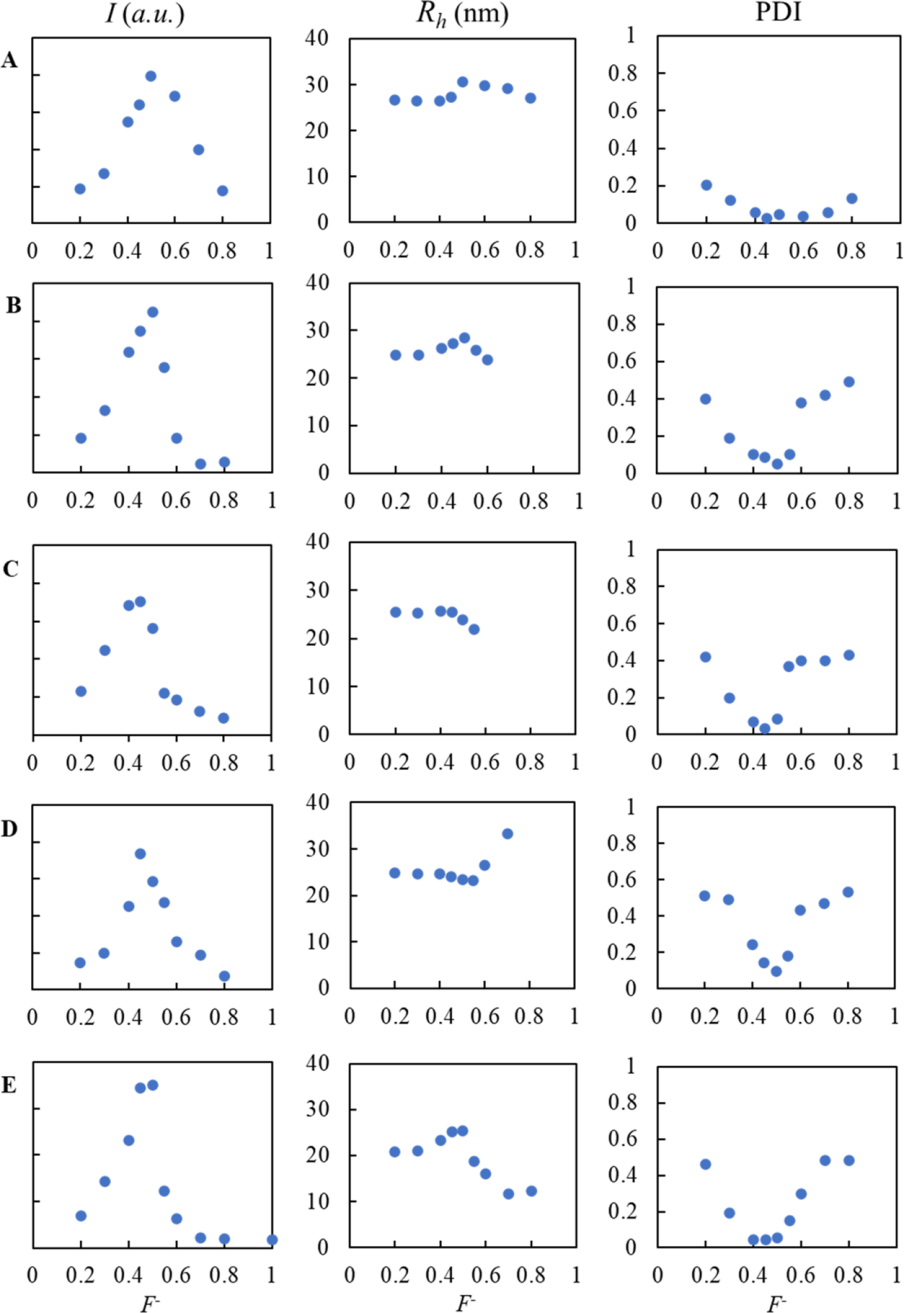
Light scattering intensity (*I*), hydrodynamic radius (*R*_h_), and polydispersity index (PDI) for mixtures of different CotA variants with the diblock copolymer PM2PV_128_-*b*-PEO_477_ observed with DLS. (A) Native CotA, (B) CotA-E10, (C) CotA-E20, (D) CotA-E30, and (E) CotA-E40. The maximum in light scattering intensity as a function of *F*^–^ corresponds to the preferred micellar composition (PMC).

In this study, we encapsulated CotA and the higher-charged CotA variants using a cationic-neutral hydrophilic diblock copolymer, PM2VP_128_-*b*-PEO_477_. For each CotA variant, several mixed ratio compositions (*F*^–^) were prepared with a constant concentration of enzyme and an increasing concentration of diblock copolymer. The different mixtures were analyzed with DLS (Figure 3). The negatively charged enzymes and the positively charged block of PM2VP_128_-*b*-PEO_477_ bind electrostatically, which is the foundation to generate C3Ms.^1,4,6,31−33^ When the charge ratio between enzyme and PM2VP_128_-*b*-PEO_477_ is about equal, the number of C3Ms in solution is expected to be highest. This composition is known as the preferred micellar composition (PMC) and is manifested by a maximum in light scattering intensity. Figure 3 shows that the PMC for native CotA and all CotA variants is indeed observed at a mixing composition (*F*^–^) of about 0.5. At the PMC, the C3Ms containing native CotA have a hydrodynamic radius (*R*_h_) of 32.0 ± 3.1 nm. For the C3Ms containing CotA with additional charges, this is 31.2 ± 1.3, 28.4 ± 1.3, 25.9 ± 2.3, and 25.4 ± 2.1 nm for CotA-E10, CotA-E20, CotA-E30, and CotA-E40, respectively. The decrease in micellar size with an increasing charge on the enzyme probably results from the fact that less enzyme molecules are needed to neutralize the charge of the diblock molecules and therefore the number of CotA molecules within one micelle is lower. At the PMC, all C3Ms show a minimum in the polydispersity index (PDI) compared to other mixing compositions. This indicates a narrow size distribution of the C3Ms around the PMC. At other mixing compositions, charged soluble complexes have been formed, resulting in lower light scattering intensities and higher PDI values.^1,4−6,8,34,35^

**Figure 4:**
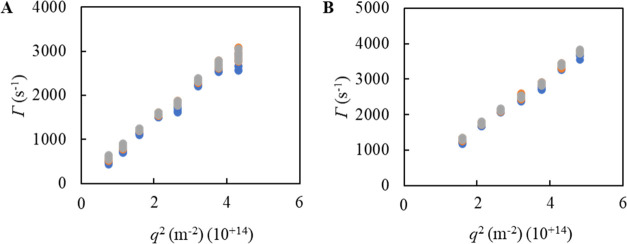
Multiangle DLS results of C3M solutions containing (A) native CotA and (B) CotA-E40. The decay rate Γ obtained from the DLS correlation curves by a first (blue), second (orange), and third (gray) cumulant fit with squared wave vector *q*^2^.

We conducted multiangle DLS to determine the shape of CotA-containing C3Ms. If C3Ms have a spherical shape, multiangle DLS will result in a linear relationship between the squared wave vector (*q*^2^) and the decay rate (Γ).^36,37^Figure 4 shows the decay rate (Γ) of the first, second, and third cumulants as a function of the squared wave vector (*q*^2^) on C3Ms composed of native CotA (Figure 4A) and CotA-E40 (Figure 4B). Three overlapping straight lines were obtained, indicating that from native CotA and CotA-E40 variants C3Ms with a spherical shape are formed.^36,37^ Similar results were found for C3Ms composed of CotA-E10, CotA-E20, and CotA-E30 variants (Figure S7, Supporting Information).

### Higher-Charged CotA Variants Show Improved C3M Stability against Salt

The addition of salt to a solution of enzyme-containing C3Ms will decrease the entropy gain of counterion release and at the same time will weaken the electrostatic interaction between the enzyme and polymer resulting in the disintegration of micelles.^1,4−6,14,35,38,39^ To reveal if the additional charges fused at the C-terminus of CotA affect C3M stability, we performed DLS measurements monitoring the scattering intensity versus salt concentration.

**Figure 5:**
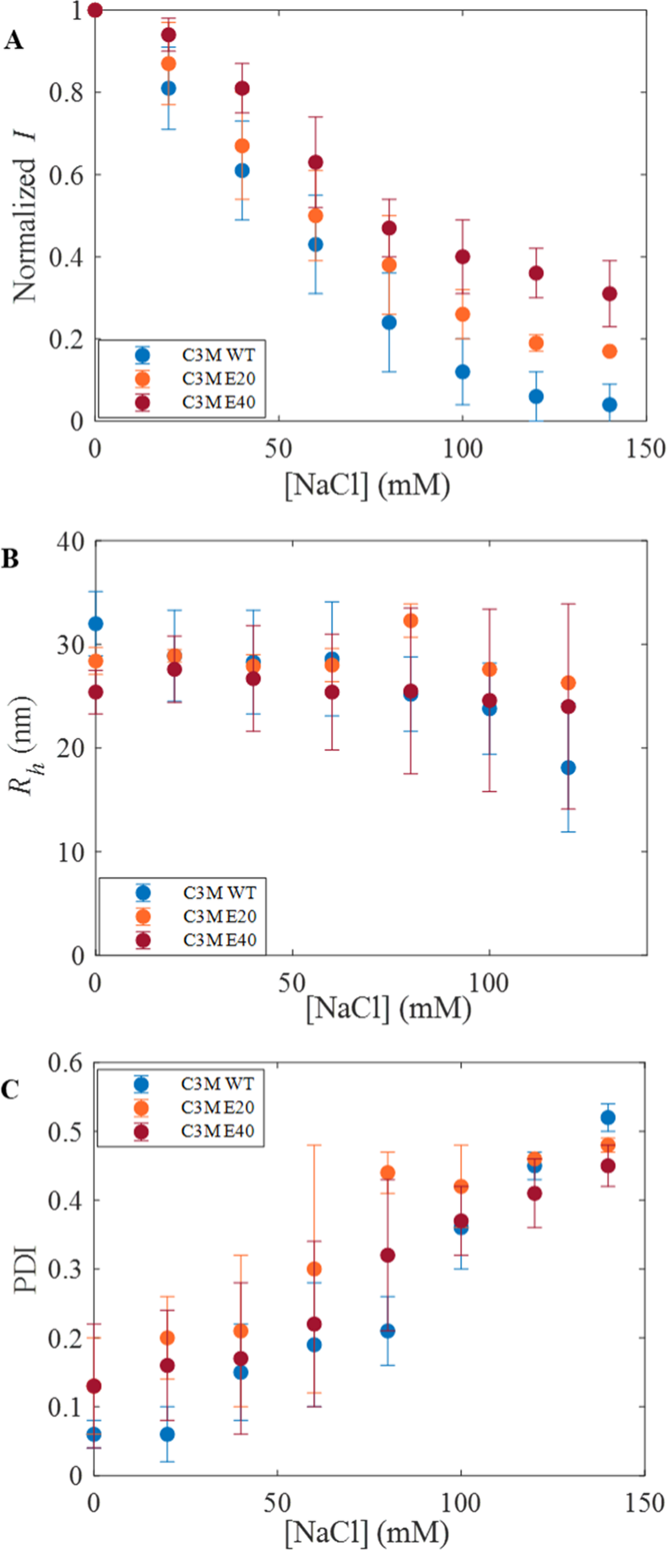
Salt stability of enzyme-containing C3Ms was observed using DLS. (A) Normalized light scattering intensity (*I*), (B) hydrodynamic radius (*R*_h_), and (C) polydispersity index (PDI). C3Ms composed of native CotA (blue), CotA-E20 (orange), and CotA-E40 (dark red). Error bars represent the standard deviation from three repetitions (*n* = 3).

Figure 5A shows the scattering intensity, normalized to its value at zero-added salt, against NaCl concentration for C3Ms composed of native CotA, CotA-E20, and CotA-E40 (see Figure S8 in the Supporting Information for the results for all CotA variants). For C3Ms composed of native CotA, the addition of salt results in a faster decline of light scattering intensity compared to C3Ms composed of additionally charged CotA variants. At a concentration of 140 mM NaCl, the normalized scattering intensity is zero, meaning there are no native CotA-C3Ms present anymore. Increasing the negative charge of CotA results in an improved salt stability of the C3Ms. Notably, the more negative charge is added to CotA, the better the resistance of the C3Ms against salt.

Differences in the salt stability of C3Ms containing CotA variants are also reflected by their difference in hydrodynamic radii (*R*_h_) as a function of NaCl concentration. Figure 5B shows that the *R*_h_ of C3Ms composed of native CotA decreases significantly with increasing NaCl concentration. After the addition of 100 mM NaCl, the size of the micelles was reduced by about 25%. However, for the higher-charged CotA variants CotA-E20 and CotA-E40, the size of the C3Ms remained fairly constant (reduction of about 3%).

During the addition of NaCl to the C3M samples, also an increase in PDI was observed, ranging from 0.1 at zero salt (monodisperse) to values above 0.4 (highly polydisperse) at 140 mM NaCl (Figure 5C). We observed that at the higher salt concentrations, not only the particle size distributions were broadened but they also showed multiple peaks. These findings indicate the disintegration of the micelles and the release of the enzyme from the micellar cores. Cumulant fitting on such heterogeneous/polydisperse samples may result in inaccurate *R*_h_ values.

### C3M Formation and Salt Stability Observed with FCS

Micelle formation and the effect of increasing the net charge of CotA on the salt stability of enzyme-containing C3Ms were also monitored by FCS. FCS and DLS are based on similar principles, but the main difference is the ability of FCS to discriminate between free enzymes in solution and encapsulated enzymes. However, for FCS, CotA needs to be labeled with a fluorescent probe, for which we used Alexa Fluor 488.

**Figure 6:**
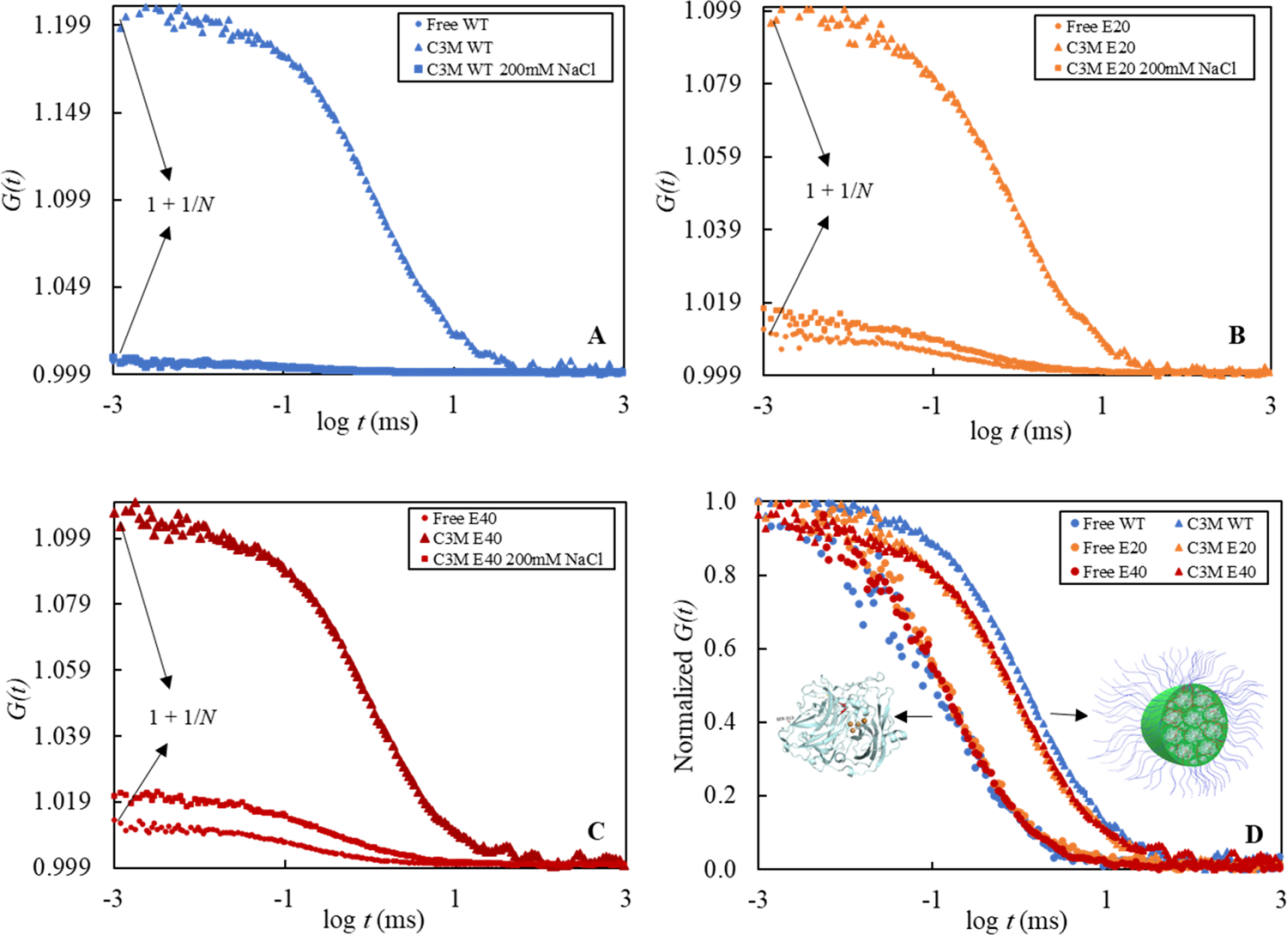
FCS measurements on C3Ms composed of labeled enzymes. FCS autocorrelation curves (*G*(*t*)) for (A) native CotA (blue), (B) CotA-E20 (orange), and (C) CotA-E40 (dark red). The spheres represent the free enzyme, the triangles represent enzyme-containing C3Ms, and the squares represent enzyme-containing C3Ms with an additional 200 mM NaCl. (D) Normalized *G*(*t*) for free enzymes and C3M samples of native CotA and higher-charged CotA variants.

Figure 6 shows FCS measurements on solutions of free CotA and CotA encapsulated in C3Ms. It becomes clear that the addition of diblock copolymer to the CotA solution shifts the autocorrelation function to a higher diffusion time, indicating that the diffusion of enzymes becomes slower due to the formation of C3Ms (Figure 6D). Moreover, FCS allows us to quantify the number of fluorescent species in the confocal volume indicated at the intercept of the Y-axis (Figure 6A–C). Encapsulation of CotA results in a decrease in the number of fluorescent particles detected in the confocal volume (*N*). Furthermore, the brightness of the micelles increases with the number of fluorescent CotA molecules encapsulated.^4,5,22,40^ The autocorrelation functions were analyzed using a two-component 3D diffusion model including triplet state (eq 8). The fraction of encapsulated enzyme was found to be about 77 ± 4, 77 ± 1, and 75 ± 7% for native CotA, CotA-E20, and CotA-E40, respectively. These results show that the encapsulation efficiency for all enzymes is approximately the same. However, the concentration of C3Ms composed of the higher-charged CotA variants is higher than that of the native CotA-containing C3Ms, illustrated by the higher number of fluorescent particles detected in the confocal volume (*N*) (Table 1).

**Table 1:** Characterization of C3Ms by FCS and DLS^a^

sample	*N* (FCS)	fraction encapsulated (FCS)	*R*_h_ (nm) (FCS)	*R*_h_ (nm) (DLS)
C3M Native CotA	4.9 ± 0.1	77 ± 4%	30.5 ± 2.1	32.0 ± 3.1
C3M CotA-E20	6.5 ± 3.6	77 ± 1%	26.0 ± 1.3	28.4 ± 1.3
C3M CotA-E40	10.7 ± 1.5	75 ± 7%	22.3 ± 1.6	25.4 ± 2.1

a Error margins represent the standard deviation from three repetitions (*n* = 3).

Furthermore, for native CotA free in solution, FCS analysis revealed an *R*_h_ of about 2.2 ± 0.3 nm, and for C3Ms containing native CotA, an *R*_h_ of 30.5 ± 2.1 nm was obtained. Free CotA-E20 and CotA-E40 have a size of 3.5 ± 0.4 and 3.1 ± 1.6 nm, respectively, whereas the *R*_h_ of micelles formed from CotA-E20 and CotA-E40 is 26.0 ± 1.3 and 22.3 ± 1.6 nm, respectively. Micellar sizes determined with FCS and DLS are in good agreement (Table 1). In conclusion, more charge added to CotA results in a higher concentration of micelles of smaller size, while the encapsulation efficiency is not significantly affected.

**Figure 7:**
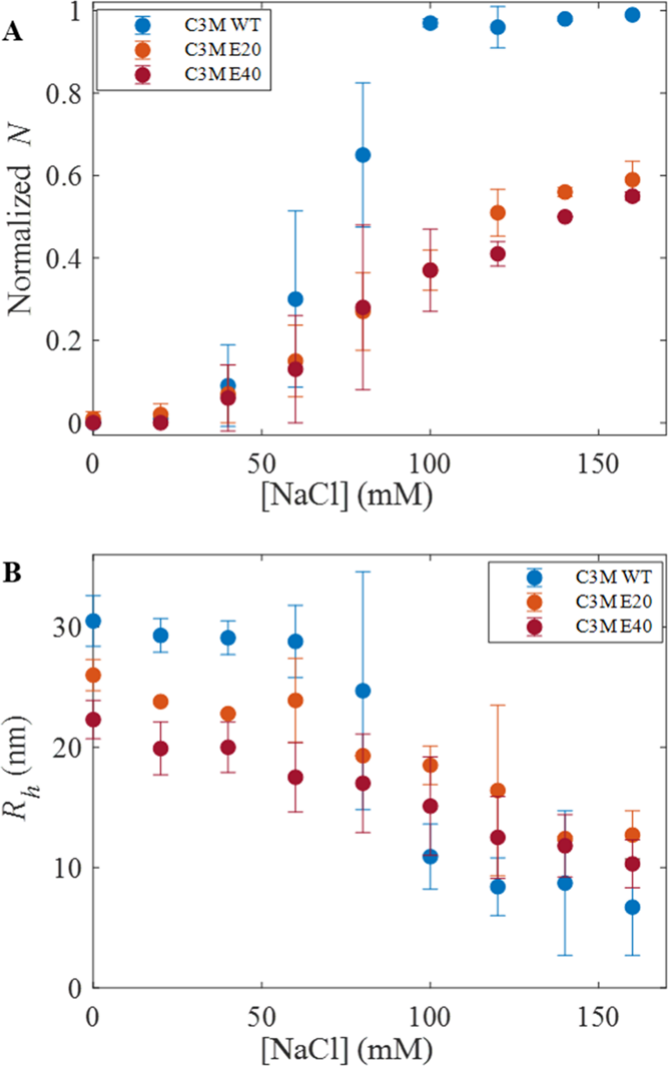
Salt stability of enzyme-containing C3Ms was observed using FCS. (A) Normalized number of fluorescent particles in the confocal volume (*N*) and (B) hydrodynamic radius (*R*_h_). C3Ms composed of native CotA (blue), CotA-E20 (orange), and CotA-E40 (dark red). Error bars represent the standard deviation from three repetitions (*n* = 3).

FCS was also used to monitor the salt stability of CotA-containing C3Ms. This was done by quantifying the total number of fluorescent particles in the confocal volume (*N*) as a function of NaCl concentration. Figure 7A shows that *N*, normalized to the number of particles detected in a solution of free CotA with the same protein concentration as the C3M systems, increases with salt concentration, which indicates the release of CotA from the core of the micelles. These results clearly show that native CotA is released at a lower salt concentration from the micelles than the higher-charged CotA variants. Native CotA-containing micelles reached a normalized *N* of 1.0 above 80 mM salt, pointing to a total disintegration of the micelles. For micelles containing CotA-E20 and CotA-E40, *N* increased much more gradually compared to micelles containing native CotA, and these C3Ms do not completely fall apart even at the highest salt concentration applied. In addition, the more negative charge is added to CotA, the better the resistance of the C3Ms against salt.

Figure 7B shows the hydrodynamic radius (*R*_h_) of the various C3Ms as a function of NaCl concentration. The *R*_h_ of C3Ms composed of native CotA decreases significantly more with increasing NaCl concentration than that of C3Ms composed of the higher-charged CotA variants: after the addition of 100 mM NaCl, the size of the native CotA micelles was decreased by about 64%, while the reduction in *R*_h_ for CotA-E20 and CotA-E40 was about 29 and 33%, respectively. These FCS measurements confirm the conclusions from the DLS measurements that the addition of polyglutamic acid tags to CotA results in improved salt stability of CotA-containing C3Ms and that the salt stability of the C3Ms increases with an increasing charge on the CotA variants.

Quenching of fluorescence upon enzyme encapsulation was observed by a reduced fluorescence intensity (Figure S9, Supporting Information).^4,5,7^ However, this quenching has no implication for the present study since we use only the number of fluorescent objects (C3Ms and free protein) in the confocal volume and their diffusion times to calculate their hydrodynamic size.

### Comparison of FCS and DLS Results

Although the DLS and FCS results show the same trends and lead to the same conclusions, there are quantitative differences in the values obtained for the mean hydrodynamic radius *R*_h_. As long as the particle size distribution in the samples is relatively narrow (before the addition of salt), FCS and DLS measurements on *R*_h_ are in good agreement (Table 1). However, after the addition of salt, the results start to deviate. Especially above a concentration of 50 mM NaCl, the *R*_h_ values obtained from FCS clearly decrease faster with the addition of salt than those obtained with DLS. Under these conditions, we are dealing with a mixture of free enzyme molecules, C3Ms, and/or soluble complexes. Thus, with the addition of salt, a broadening of the particle size distribution and the development of multiple peaks takes place, reflected in increasing PDI values. These changes in the particle size distribution can explain the observed deviations in *R*_h_ values from DLS and FCS as follows. In DLS, the scattered intensity of small particles (i.e., the free enzymes) is negligible with respect to that of the larger particles (C3Ms and soluble complexes), so the calculated *R*_h_ value is dominated by the contribution of the C3Ms and soluble complexes.^28,41^ On the other hand, in FCS, every fluorescent particle in the confocal volume contributes equally to the determination of the average diffusion time and thus to the mean hydrodynamic radius (see eqs 8, 9, and 5). This leads to lower *R*_h_ values than those following the DLS analysis.^28,41^ Hirschle et al. also observed larger average particle sizes by using DLS compared to FCS on broader size distribution samples.^28^ Moreover, at very low scattered light intensities, *R*_h_ determination using DLS becomes inaccurate. Overall, the *R*_h_ results of FCS are considered more reliable.

Finally, we note that the deviations in the *R*_h_ values may also be partly due to the different fitting models used in DLS and FCS. In DLS, the autocorrelation function was fitted using the cumulant method, while in FCS, the autocorrelation function was fitted using a two-component model.

### Effect of Genetic Modification and Encapsulation on the Activity of CotA

**Figure 8:**
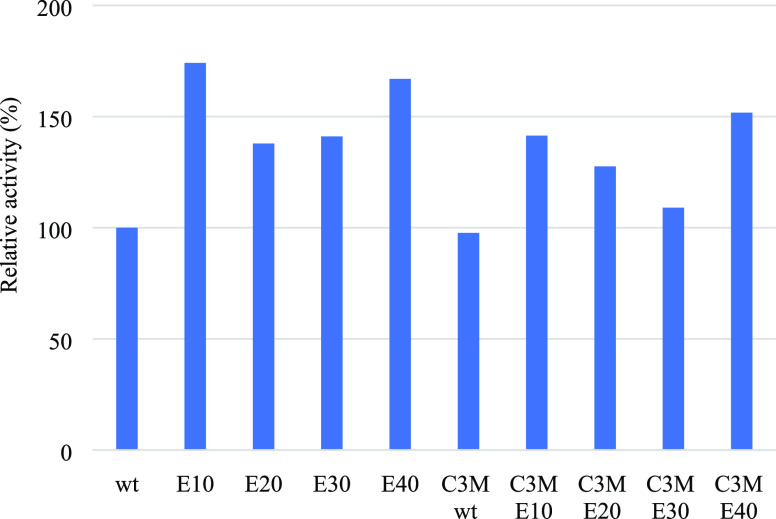
Activity measurement of native CotA (CotA-WT), CotA-E10, CotA-E20, CotA-E30, and CotA-E40 and its C3Ms.

Enzyme activity measurements were conducted to evaluate whether the addition of charged amino acids influences the enzyme function. Here, CotA activity measurements were done with the substrate ABTS (for details, see the Experimental Section). Figure 8 shows the relative activity histogram for native CotA and CotA variants free in solution as well as after being encapsulated in C3Ms. The activity of native CotA free in solution is set to 100%. Remarkably, the higher-charged CotA variants showed significantly higher activities than native CotA: their relative activities amount to about 170 ± 1.1, 140 ± 0.5, 140 ± 0.5, and 160 ± 1% for CotA-E10, CotA-E20, CotA-30, and CotA-40, respectively. A possible explanation for the enhanced activity of the charged variants is the improvement of their solubility.^10,45−48^ Han et al. also found an increased solubility and catalytic activity of several enzymes (tyrosine ammonia lyase, aldehyde dehydrogenase, and 1-deoxy-d-xylulose-5-phosphate synthase) after the addition of short polyglutamic acid tags.^10^

Next, we aimed at measuring the activity of CotA when encapsulated in C3Ms also with the substrate ABTS. However, this was not possible since the ABTS assay procedure involves a low pH buffer (pH 4.4), resulting in a positive net charge on the enzyme (pI at pH 5.84 for native CotA) and therefore disintegration of the C3Ms. C3Ms composed of higher-charged CotA variants also disintegrate at this pH (Figure S10, Supporting Information). Figure 8 shows that encapsulation and subsequent release in the ABTS assay buffer do not affect the activity of native CotA. For the higher-charged CotA variants, the activity decreases somewhat as a result of encapsulation and pH-induced release, but it is still higher than that of native CotA free in solution.

## Conclusions

In this study, we produced variants of CotA that have polyglutamic acid tags of different lengths at the C-terminus. These polyglutamic acid tags contribute to a higher negative charge of the enzyme and are therefore expected to increase the salt stability of C3Ms containing the enzyme. DLS measurements revealed that for all CotA variants the PMC for C3M formation with the cationic-neutral-hydrophilic diblock copolymer PM2PV_128_-*b*-PEO_477_ is at a mixing composition (*F*^*–*^) of about 0.5. FCS measurements showed that increasing the net charge of CotA results in the formation of more C3Ms but with a lower number of CotA molecules within one micelle. The overall encapsulation of the native and modified enzymes was approximately the same, i.e., 75–77% of the enzyme molecules was incorporated in the micelles. DLS and FCS measurements confirmed that C3Ms composed of CotA with additional charges are significantly more salt-resistant than native CotA-containing micelles. The more extra charge is added to the CotA, the better the salt stability of C3Ms. Furthermore, it was found that adding a polyglutamic tag to the enzyme enhances its activity, which is largely maintained upon encapsulation and pH-induced release from the C3Ms. This study therefore shows that increasing the net charge of enzymes by genetic engineering is a promising strategy to improve the practical applicability of C3Ms as enzyme delivery systems.

## Supporting Information Available

(1) ^1^H NMR spectrum of PM2PV_128_-*b*-PEO_477_; (2) primer sequences for cloning; (3) agarose gel electrophoresis results on modified CotA; (4) charge of CotA as a function of pH; (5) color of the solutions of purified native CotA and the glutamic acid CotA variants; (6) DLS multiangle results on C3Ms containing higher-charged enzyme variants; (7) DLS results on salt stability of enzyme-containing C3Ms for all C3M samples; (8) fluorescence spectra of labeled enzymes free in solution and encapsulated in C3Ms; (9) DLS results on the pH stability of enzyme-containing C3Ms; (10) CotA activity measurements (PDF)
