# Supplementary material for: Charged Polypeptide Tail Boosts the Salt Resistance of Enzyme-Containing Complex Coacervate Micelles
Source: Biomacromolecules. 2022 Jan 19;23(3):1195–204. doi: 10.1021/acs.biomac.1c01466 (PMC8924873; doi:10.1021/acs.biomac.1c01466)
Supplement: Supplementary file 1 — bm1c01466_si_001.pdf [file bm1c01466_si_001.pdf]

**Supporting Information**

# Charged Polypeptide Tail Boosts the Salt Resistance of Enzyme-Containing Complex Coacervate Micelles

*Riahna Kembaren*[a,b]*, Adrie H. Westphal*[b]*, Marleen Kamperman*[c]*, J. Mieke Kleijn*[a]*, Jan Willem Borst*[b]

[a]Physical Chemistry and Soft Matter, Wageningen University & Research, Stippeneng 4, 6708 WE Wageningen, The Netherlands.

[b]Laboratory of Biochemistry, Microspectroscopy Research Facility, Wageningen University & Research, Stippeneng 4, 6708 WE Wageningen, The Netherlands.

[c]Polymer Science, Zernike Institute for Advanced Materials, University of Groningen, Nijenborgh 4, 9747 AG Groningen, The Netherlands.

**$^{1}$H-NMR Spectrum of the Quaternized Diblock Copolymer**

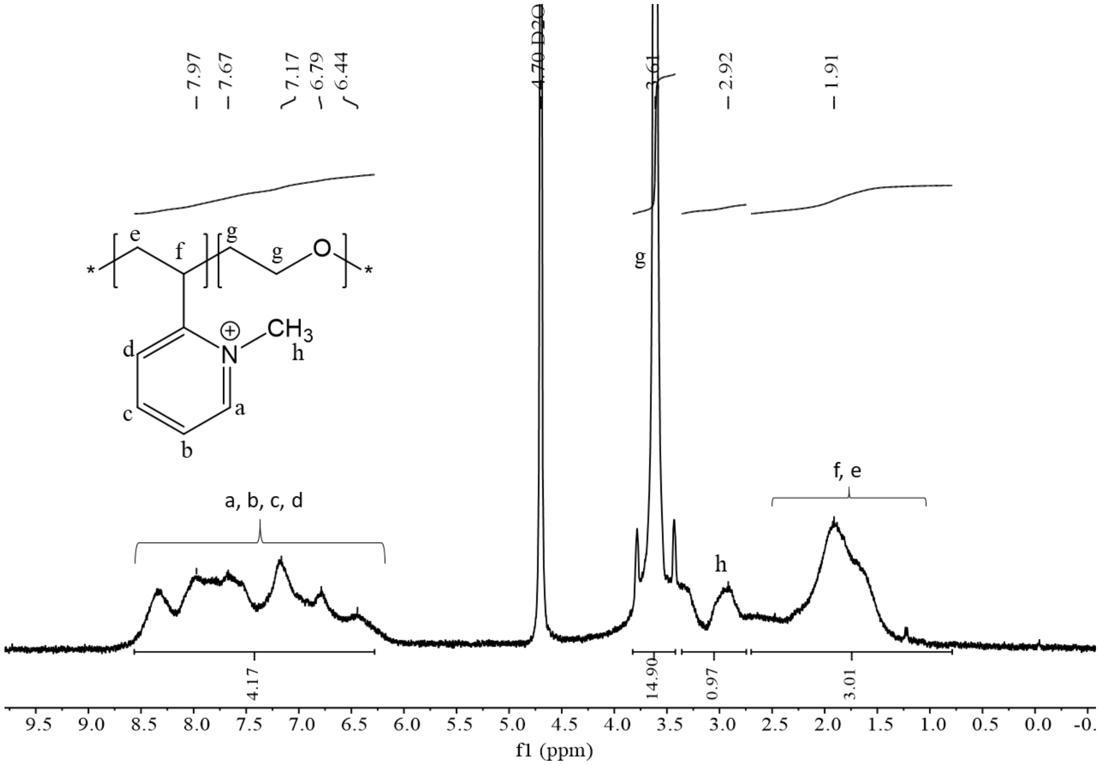


**Figure S1**. $^{1}$H-NMR spectrum of $PM2PV_{128}$-*b*-$PEO_{477}$ was recorded in $D_2O$. The degree of quaternization (DQ) was calculated by comparing the sum integral of the pyridine ring peak with that of the methyl group.

DQ = ((4.17 + 0.97) / (4+3)) *100% = 73%.

**Primer Sequences for Cloning**

Primers that were used to add polyglutamic acid tags were purchased from IDT. For PCR, the template that has been used is the native CotA gene. The primer sequences used are listed below:

a. Forward primer (for all engineered CotA): CotA-N-forward 5'-
GGCTAACAGGAGGAATTACATATGACACTTGAAAAATTTGTGGATGCTC

b. Reverse primer for Native CotA: CotA-wt-rev 5'-
GAGTTTTTGTTCGGGCCCAAGCTTTTATTTATGGGGATCAGTTATATCCATCGG

c. Reverse primer for CotA-E10: CotA-E10-Rev 5’-
GAGTTTTTGTTCGGGCCCAAGCTTTTATTCCTCTTCTTCCTCTTCTTCCTCTTCTTCTTT
ATGGGGATCAGTTATATCCATCGG

d. Reverse primer for CotA-E20: CotA-E20-Rev 5'-
GAGTTTTTGTTCGGGCCCAAGCTTTTATTCTTCCTCTTCTTCCTCTTCTTCCTCTTCTTC
CTCTTCTTCCTCTTCTTCCTCTTCTTCTTTATGGGGATCAGTTATATCCATCGG

e. Reverse primer for CotA-E30: CotA-E30-Rev 5'-
GAGTTTTTGTTCGGGCCCAAGCTTTTACTCTTCTTCCTCTTCTTCCTCTTCTTCCTCTTC
TTCCTCTTCTTCCTCTTCTTCCTCTTCTTCCTCTTCTTCCTCTTCTTCCTCTTCTTCTTTA
TGGGGATCAGTTATATCCATCGG

f. Reverse primer for CotA-E40. CotA-E40-Rev 5' –
GAGTTTTTGTTCGGGCCCAAGCTTTTATTCCTCTTCTTCCTCTTCTTCCTCTTCTTCCTC
TTCTTCCTCTTCTTCCTCTTCTTCCTCTTCTTCCTCTTCTTCCTCTTCTTCCTCTTCTTCC
TCTTCTTCCTCTTCTTCCTCTTCTTCTTTATGGGGATCAGTTATATCCATCGG

## Agarose Gel Electrophoresis

CotA-E10

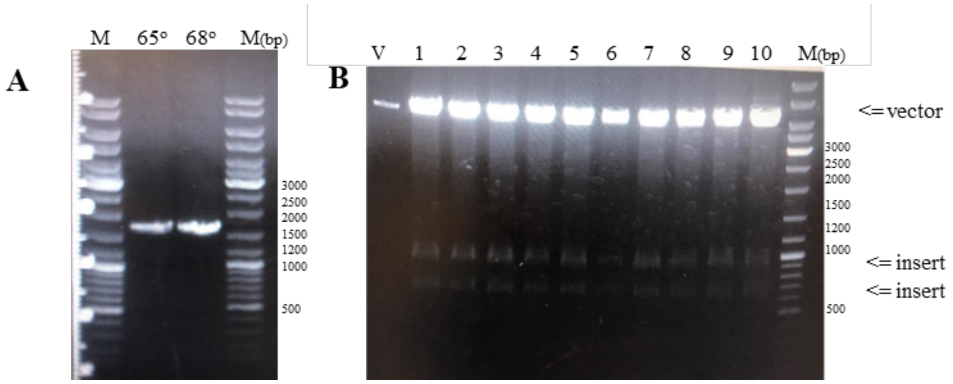


**Figure S2.** Agarose gel electrophoresis results for CotA-E10. (A) After running PCR, (B) after digestion using restriction enzymes *Nde*I and *Hin*dIII on the isolated plasmid after cloning and transformation. V is vector, M are markers.

Figure S3A shows the successful modification of the CotA with 10 additional glutamic acid residues by PCR using annealing temperatures of 65 ºC or 68 ºC. Figure S3B shows that 10 randomly selected *E. coli* colonies contain the inserted gene of CotA-E10.

CotA-E20

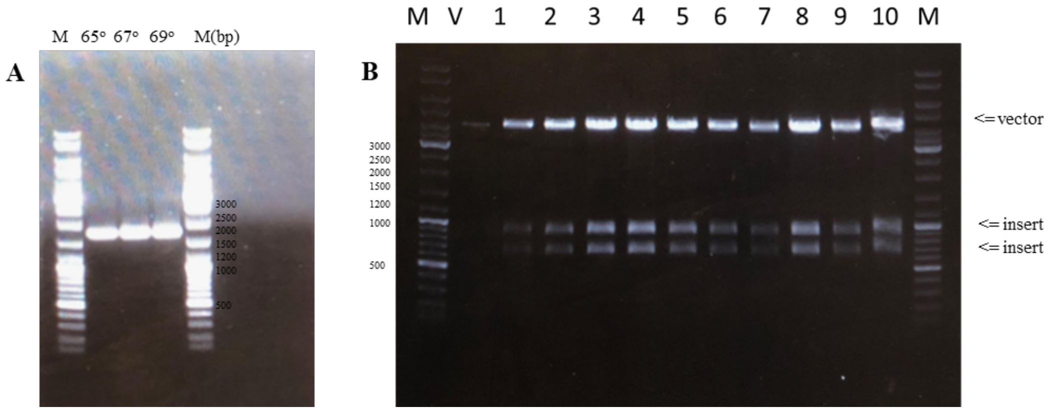


**Figure S3.** Agarose gel electrophoresis results for CotA-E20. (A) After running PCR, (B) after digestion using restriction enzymes *Nde*I and *Hin*dIII on the isolated plasmid after cloning and transformation. V is vector, M are markers.

Figure S4A shows the successful modification of the CotA with 20 additional glutamic acid residues by PCR using annealing temperatures of 65 ºC or 67 ºC or 69 ºC. Figure S4B shows that 10 randomly selected *E. coli* colonies contain the inserted gene of CotA-E20.

CotA-E40

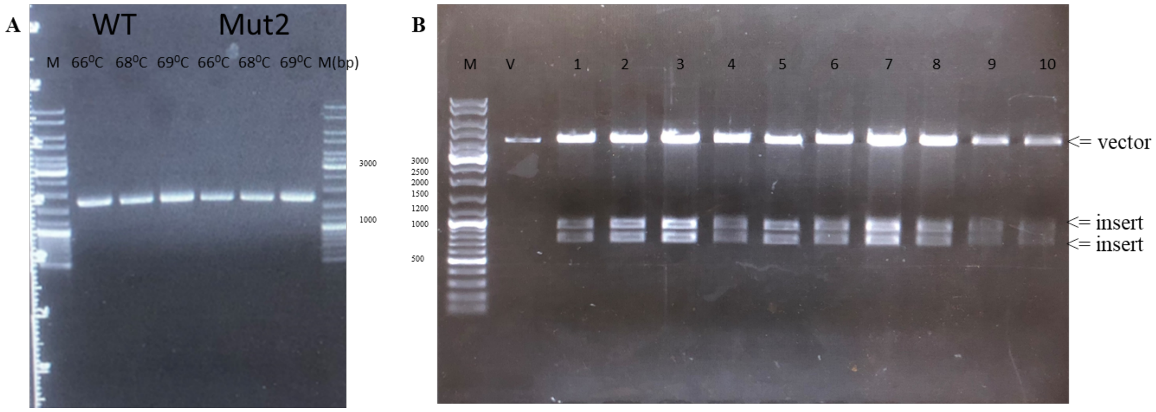


**Figure S4.** Agarose gel electrophoresis results for CotA-E40. (A) After running PCR, (B) after digestion using restriction enzymes *Nde*I and *Hin*dIII on the isolated plasmid after cloning and transformation. V is vector, M are markers.

Figure S5A shows the successful modification of the CotA with 40 additional glutamic acid residues by PCR using annealing temperatures of 66 ºC or 68 ºC or 69 ºC. Figure S5B shows that 10 randomly selected *E. coli* colonies contain the inserted gene of CotA-E40.

**Calculated Charge of CotA as a Function of pH**

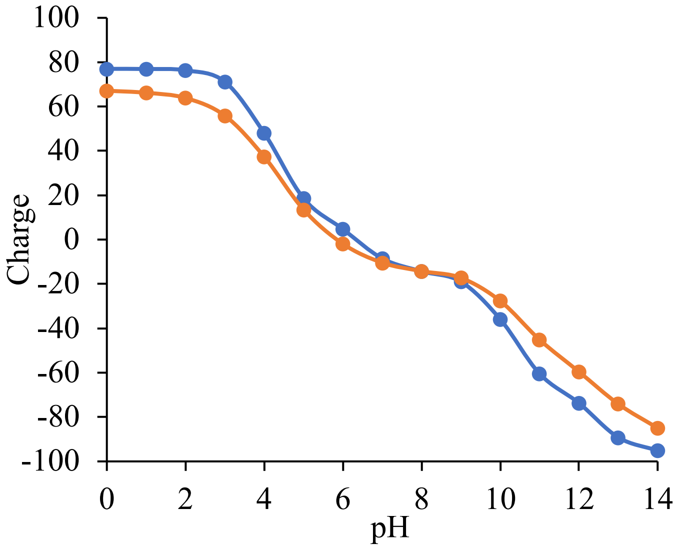


**Figure S5**. Charge of CotA as a function of pH calculated by using the PROPKA 3.1 software package. Blue dots and line represent the unfolded state of CotA, and orange dots and line represent the folded state of CotA.

**Color of the solutions of purified Native CotA and the Glutamic Acid CotA Variants**

A

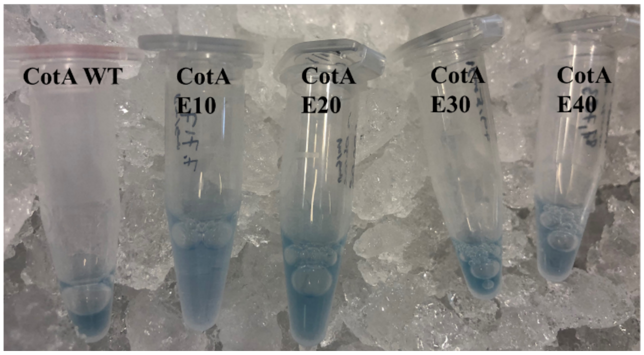


B

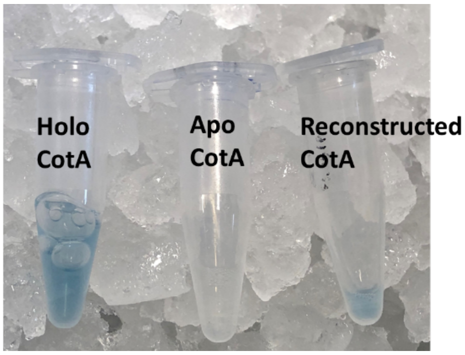


**Figure S6.** The color of the enzyme solutions: (A) the purified native CotA and the glutamic acid CotA variants (CotA-E10, CotA-E20, CotA-30, and CotA-E40). (B) holoprotein, apoprotein and reconstructed protein. The blue color (absent in apo CotA) is a sign of the copper ion (T1 Cu ion) incorporated in the protein resulting in active enzyme.

**DLS Multiangle Results on C3Ms Containing Higher Charged Enzyme Variants**

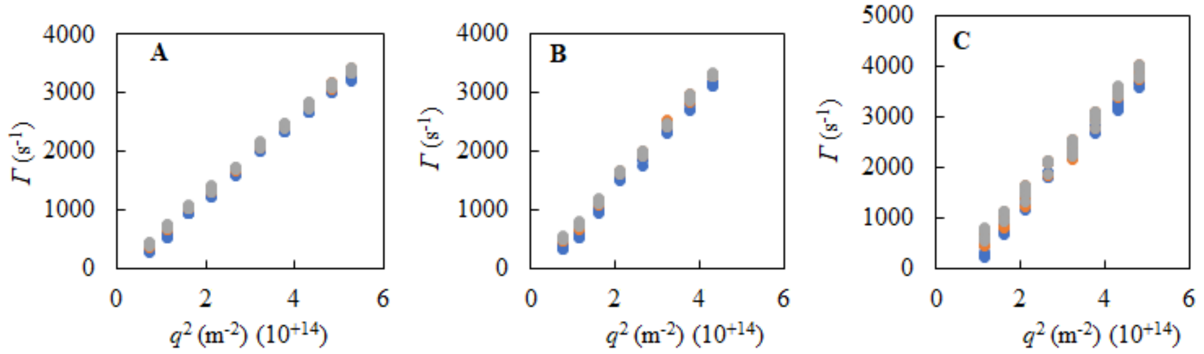


**Figure S7.** Multiangle DLS results of C3M solutions containing (A) CotA-E10, (B) CotA-E20, (C) CotA-E30. The decay rate *Γ* obtained from the DLS correlation curves by a first (blue), second (orange), and third (gray) cumulant fit with squared wave vector $q^2$.

## DLS Results on Salt Stability of Enzyme-Containing C3Ms for All C3Ms Samples

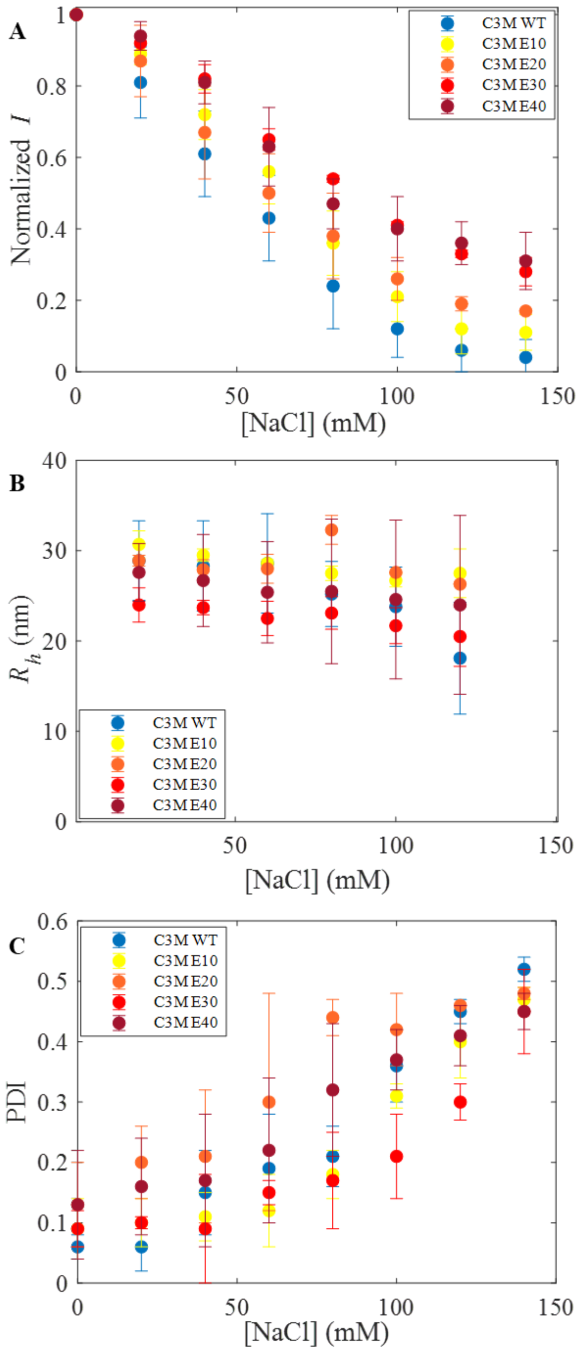


**Figure S8.** Salt stability of enzyme-containing C3Ms observed by using DLS. (A) Normalized light scattering intensity (*I*), (B) hydrodynamic radius ($R_h$), (C) polydispersity index (PDI). C3Ms composed of native CotA (blue), CotA E-10 (yellow), CotA E-20 (orange), CotA E-30 (red), and CotA-E40 (dark red). Error bars represent the standard deviation from three repetitions ($n = 3$), and for each of these repetitions the result was the average of 10 measurements of 10 seconds.

**Fluorescence spectra of labeled enzymes free in solution and encapsulated in C3Ms**

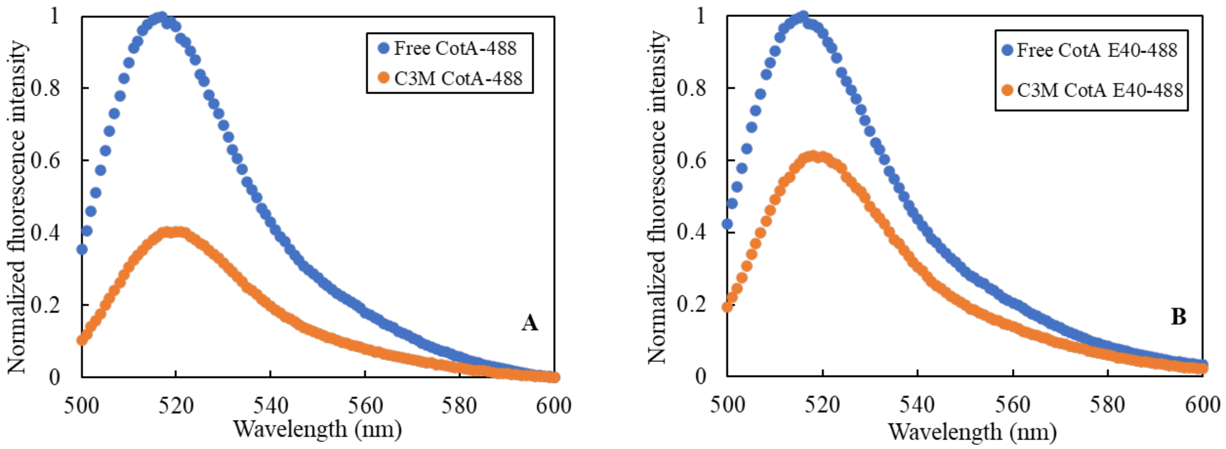


**Figure S9.** Normalized fluorescence emission spectra showing the effect of quenching upon encapsulation of CotA (A) Native CotA (CotA WT), (B) CotA-E40. Free enzyme (blue), C3Ms (orange). The fluorescence emission spectra were recorded using Cary Eclipse Spectrofluorimeter (Varian Inc.) by exciting Alexa-488 CotA at a wavelength of 490 nm and scanning the fluorescence emission from 500 to 600 nm.

**DLS Results on pH Stability of Enzyme-Containing C3Ms**

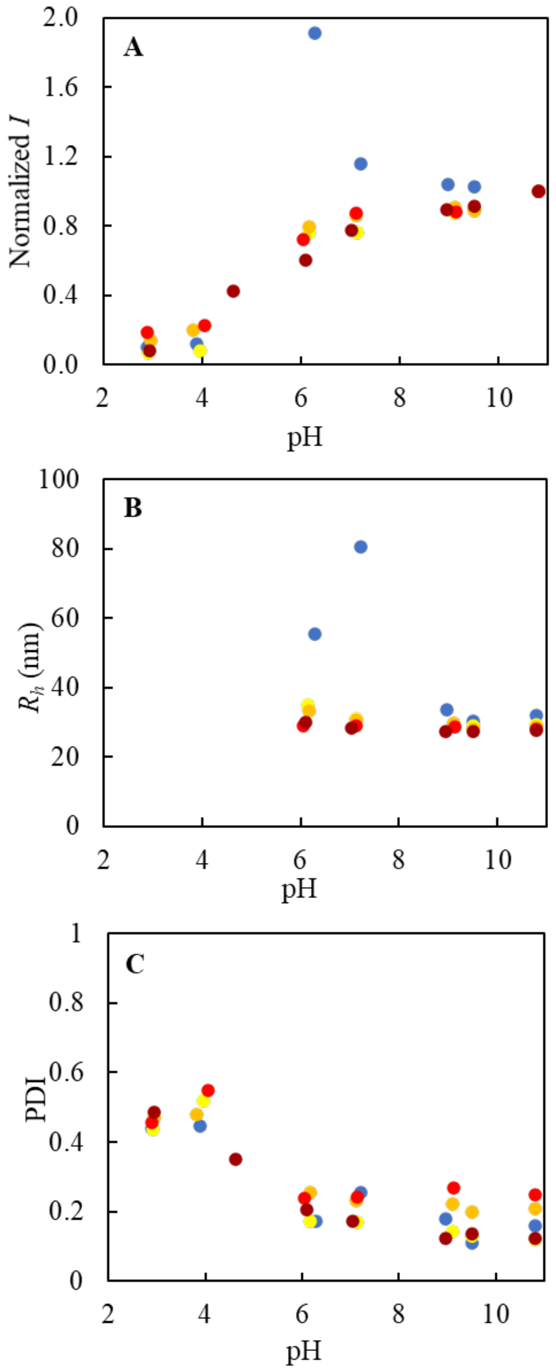


**Figure S10.** pH stability of enzyme-containing C3Ms observed by using DLS. (A) Normalized light scattering intensity (*I*)), (B) hydrodynamic radius ($R_h$), (C) polydispersity index (PDI). C3Ms composed of native CotA (blue), CotA E-10 (yellow), CotA E-20 (orange), CotA E-30 (red), and CotA-E40 (dark red).

**CotA Activity Measurements**

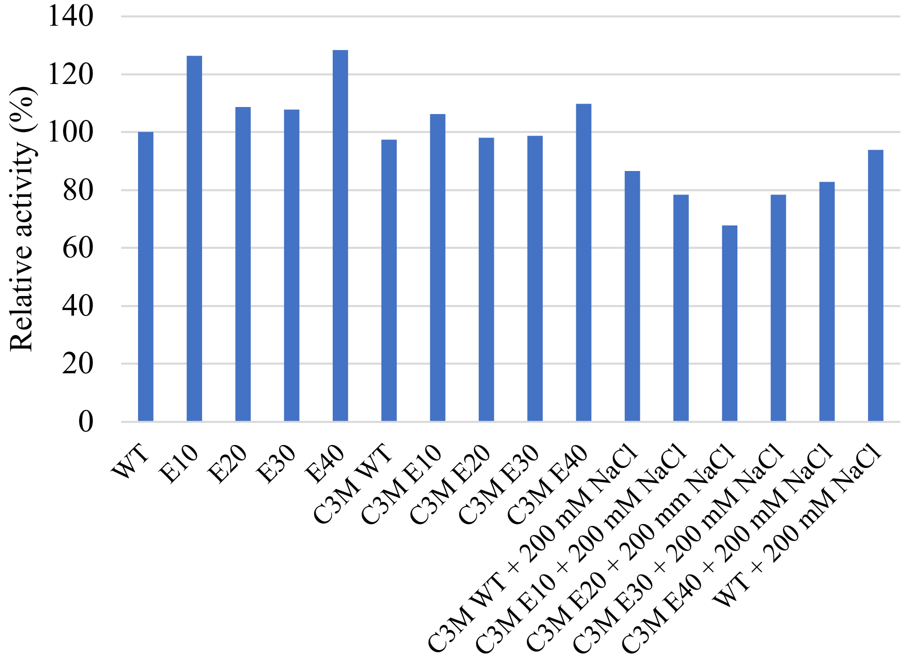


**Figure S11.** Activity measurements of native CotA (CotA-WT), CotA-E10, CotA-E20, CotA-E30, and CotA-E40 free in solution and encapsulated in C3Ms in the presence and absence of 200 mM NaCl.
